# Screening Newborns for Hearing Loss under Full Public Funding, Kochi, Japan -Differences in the Screening Results between Premature Neonates and Healthy Newborns

**DOI:** 10.31662/jmaj.2021-0203

**Published:** 2022-03-04

**Authors:** Ichiro Fukunaga, Taisuke Kobayashi, Kahori Hirose

**Affiliations:** 1 Treatment and Welfare Center, Kochi Prefectural Government, Kochi, Japan; 2 Aki Public Health and Welfare Office, Kochi Prefectural Government, Kochi, Japan; 3 Department of Otolaryngology, Head and Neck Surgery, Kochi Medical School, Kochi, Japan

**Keywords:** newborn hearing screening, congenital hearing loss, full public expenditure, hearing loss prevalence, neonatal intensive care unit

## Introduction

Congenital hearing loss (HL) occurs in approximately one to two newborns per 1000 live births ^(1), (2)^. Newborn hearing screening (NHS) can aid early detection of HL and facilitates prompt treatment and education ^(3), (4)^.

Since 2017, all municipalities of the Kochi Prefecture, Japan, include NHSs at full public expense. All testings are performed using automated auditory brainstem responses (aABRs), and medical institutions submit test results to the municipalities. The municipalities compile the results and report them to the prefectural government. Additionally, public health nurses will support families whose newborns have been required diagnostic examinations.

We report the NHS results of newborns born between April 2017 and March 2020. We also compared newborns who were screened in the neonatal intensive care unit (NICU) with those who were not. Prematurity has been reported to be associated with a higher frequency of congenital HL ^(5)^; thus, the premature equivalent should be excluded from the statistical analysis to evaluate screening in healthy newborns (HN). Additionally, we determined the differences in the screening results between premature neonates and HN.

## Methods

### Subjects

We included newborns born in the Kochi Prefecture between April 2017 and March 2020. In Kochi Prefecture, HN were initially screened using aABR on postnatal days 2-4 (first screening). In the first screening, only if both ears passed the assessment, the result was classified as “pass,” and if a referral was warranted unilaterally or bilaterally, the result was classified as “refer,” and rescreening was performed. During the rescreening, as in the first screening, both ears were tested again using aABR, and if the result was classified as “refer,” the diagnostic examination was requested. Newborns weighing ≥1500 g at birth with no obvious chromosomal abnormalities receive aABR in the NICU. Preterm newborns receive aABR after 35 weeks’ gestation.

Each fiscal year, the municipalities submit aggregate NHS results to the prefectural government for newborns born between April 1 (previous year) and March 31 (current year). Three hospitals with NICUs also submit aggregate NHS results for newborns born during the same period and those admitted to the NICU to the prefectural government. We aggregated and analyzed the prefectural government screening data from the 34 municipalities in Kochi Prefecture and the NICUs. Clarifications regarding the data were obtained directly from the municipality or hospital.

### Referral and prevalence rates calculations

We collected the number of births in Kochi Prefecture (i.e., the number of newborns available for screening), the first and rescreening results, the number of newborns who underwent diagnostic examination, and the diagnostic examination results. Regarding NICU data, we collected the number of NHSs performed and the first and rescreening results.

We calculated three NHS referral rate indicators: 1) first screening referral rate (%): proportion of first screenings referred for rescreening; 2) rescreening referral rate (%): proportion of rescreenings referred for diagnostic examinations; and 3) screening referral rate (%): ratio of the number of persons referred for diagnostic examinations after being rescreened to the number of individuals in the first screenings conducted.

**Figure 1.**
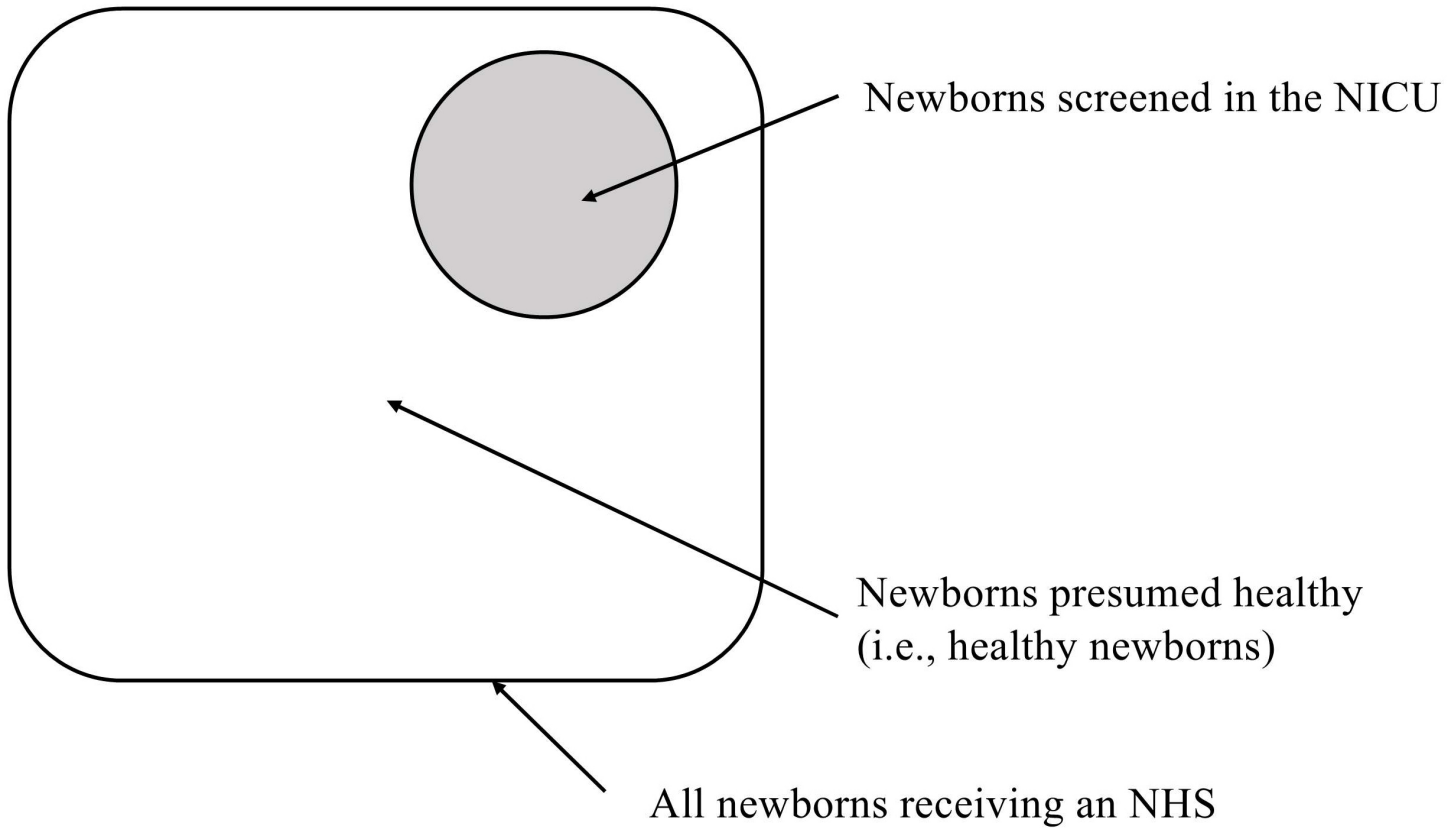
Euler diagram estimating the number of presumed healthy newborns. In the perinatal care system of the Kochi Prefecture, all premature newborns are admitted to one of three hospitals with NICUs almost without exception. In most cases, pregnant women are referred to these hospitals if the physician predicts premature birth. Thus, the newborns receiving a hearing screening and not admitted to the NICU are presumed to be healthy. Hence, subtracting the number of newborns screened in the NICU from the total number of newborns screened excludes premature newborns and estimates the number of healthy newborns. Abbreviations: NICU, neonatal intensive care unit; NHS, newborn hearing screening.

In the perinatal care system of the Kochi Prefecture, all premature newborns are admitted to one of three hospitals with NICUs almost without exception. Pregnant women are often referred to these hospitals if premature birth is likely. Thus, newborns who undergo NHS and are not admitted to the NICU are presumed to be healthy. Therefore, subtracting the number of newborns screened in the NICU from the total number of newborns screened excludes premature newborns and estimates the number of HN. Then, for each number, we subtracted the number of newborns who received screening in the NICU from the total number of newborns who received screening in Kochi Prefecture and calculated three NHS referral rate indicators. These showed the referral rate for presumed HN. We were able to compare the referral rates of newborns who were screened in the NICU (i.e., premature newborns) with those who were presumed HN. The referral rates were calculated for HNs and compared with the NICU referral rates (Figure 1).

The HL prevalence rates were calculated for the diagnostic examination results. The prevalence rates were calculated by dividing the number of HLs by the number of first screenings conducted.

### Statistical analyses

The odds ratios (ORs) of the NICU referral rate were calculated using HN as 1 with 95% confidence intervals (95% CIs). We used Chi-square tests with Yates’ correction, P-values of <0.05 were considered statistically significant. EZR (Version 1.5) ^(6)^ was used for the statistical analyses.

## Results

**Figure 2.**
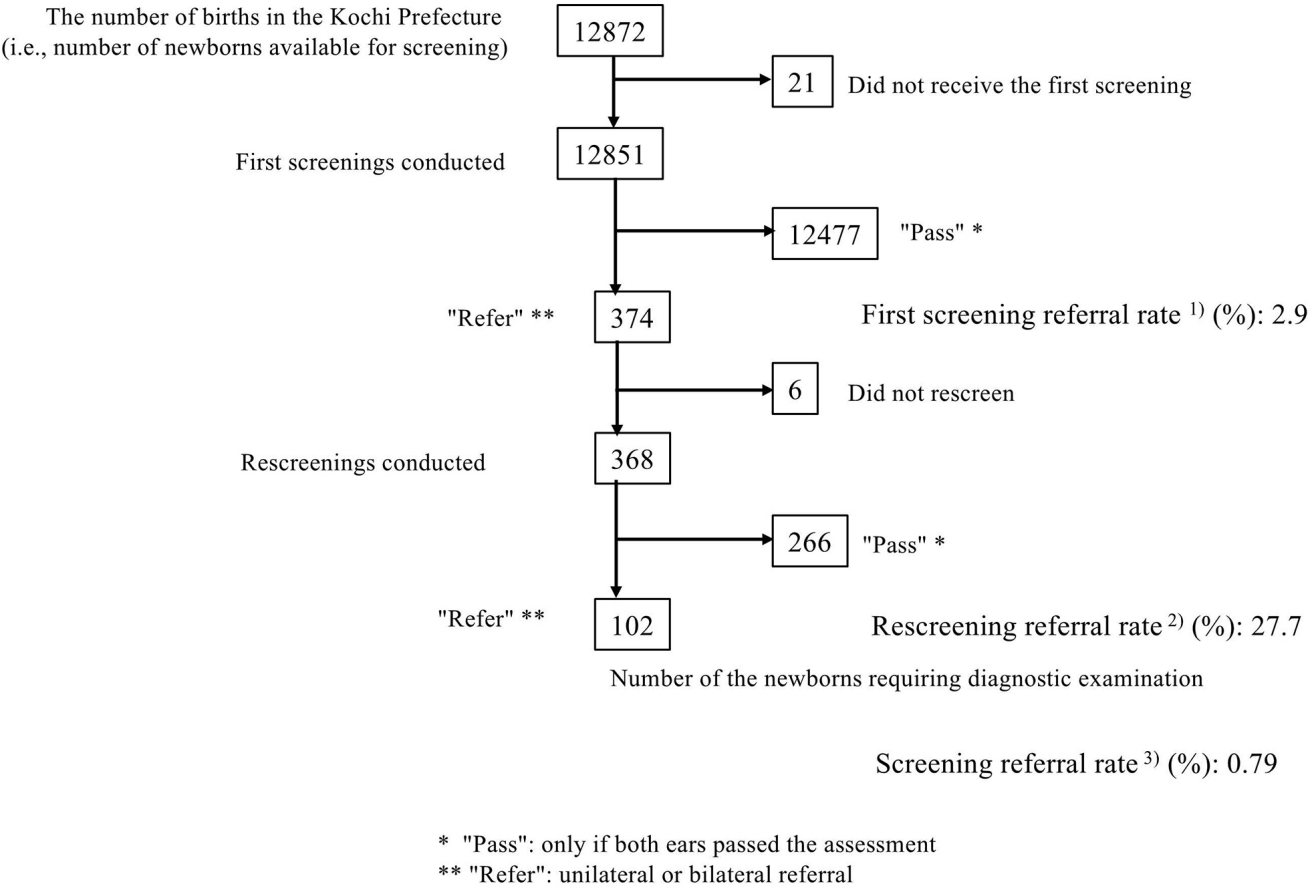
Newborn hearing screening results. Of all newborns in the Kochi Prefecture, 12872 received a hearing screening, and 21 did not. The NHS test results are classified as “pass” or “refer.” Overall, 12477 received the pass result and 374 were referred for first screenings; 368 newborns underwent rescreening (21 did not), resulting in 266 passes and 102 referrals. Overall, 0.79% of newborns required diagnostic examination. ^1)^ The proportion of first screenings referred for rescreening. ^2)^ The proportion of rescreenings referred for diagnostic examination. ^3)^ The proportion of first and rescreenings conducted that were also referred by rescreenings.

In total, 12851 newborns were screened (99.2% of all newborns). The first screening referral rate was 2.9%, the rescreening referral rate was 27.7%, and the screening referral rate was 0.79% (Figure 2). In total, 1589 newborns were screened in the NICU (12.3% of all newborns). The first screening referral rate was 6.7%, the rescreening referral rate was 39.6%, and the screening referral rate was 2.64%. We calculated that 11262 presumed HN were screened; the first screening referral rate was 2.4%, the rescreening referral rate was 22.4%, and the screening referral rate was 0.53%.

The ORs for NHS in the NICU were 2.93 (95% CI, 2.30-3.71) for the first screening referral rate and 2.27 (95% CI, 1.36-3.79) for the rescreening referral rate when the presumed HN was 1. The screening referral rate OR was 5.07 (95% CI, 3.32-7.67). Chi-square tests (Yates’ correction) demonstrated significant differences in all ORs (Table 1).

**Table 1.** Newborns Screened in the NICU versus Healthy Newborns.

Referral rate	NICU	Healthy	Odds ratio	95% CI	p-value*
First screening^1^	6.7%	2.4%			
	(106/1589)	(268/11262)	2.93	2.30-3.71	<0.001
Rescreening^2^	39.6%	22.4%			
	(42/106)	(60/268)	2.27	1.36-3.79	0.001
Screening^3^	2.64%	0.53%			
	(42/1589)	(60/11262)	5.07	3.32-7.67	<0.001

*Chi-square tests (Yates’ correction); p-values <0.05 are significant. ^1)^ Proportion of first screenings referred for rescreening. ^2)^ Proportion of rescreenings referred for diagnostic examination. ^3)^ Ratio of the number of persons referred for diagnostic examinations after being rescreened to the number of individuals in the first screenings conducted. Abbreviations: NICU, neonatal intensive care unit; CI, confidence interval.

Overall, 102 newborns received diagnostic examinations, and the results were available for 81 (79.4%); the results of the other patients were unavailable because of relocation or loss of contact. Of the available results, 17 (21.0%) had decisions withheld, 27 (33.3%) had no HL, and 37 (45.7%) had HL. Bilateral HL was present in 14 patients. Of the 12851 screened newborns, 0.29% had HL, and 0.11% had bilateral HL (Table 2).

**Table 2.** Diagnostic Examination Results.

Date of birth	2017-2018^†^	2018-2019^†^	2019-2020^†^	Total	Proportion (%)
Number of diagnostic examinations	38	34	28	102	
Final result unknown^※^	1	10	7	18	
Final result known	37	23	21	81	100.0
**Results**					
Decisions withheld^‡^	11	4	2	17	21.0
No hearing loss	15	8	4	27	33.3
Hearing loss	11	11	15	37	45.7
Bilateral	5	5	4	14	17.3
Unilateral	6	6	11	23	28.4

^※^The main reasons for an unknown result were a change of address and lost contact. ^†^From April to March. ^‡^Hearing loss could not be ruled out, and the patients were placed on observation.

## Discussion

The main causes of congenital HL are hereditary factors, in utero cytomegalovirus infection ^(7), (8)^, and auditory neuropathy ^(9)^. Hereditary HL is often inherited as an autosomal recessive trait ^(10)^ and may initially look like an isolated case. Also, the NHS referral rate is higher for newborns admitted to the NICU than for HNs ^(11)^.

This study estimated the probability of newborns being referred after screening by arithmetic calculation using two aggregate populations: the municipalities and the NICUs. The probabilities of screening referrals (i.e., screening referral rates) in Japan are reportedly 0.52% (in Okayama) ^(12)^ and 0.53% (in Akita) ^(1)^. The screening referral rate for the estimated HNs in this study was 0.53%, which agrees with existing reports.

The newborns admitted to the NICU were more likely to be referred than presumed HNs. HL prevalence at birth was predicted to be higher in newborns admitted to the NICU than in HNs. Despite the necessity of waiting for auditory conduction pathway maturation before screening ^(13), (14)^, the aABR has been widely performed in children admitted to the NICU ^(10), (15)^.

The diagnostic results showed that 45.1% of the examined newborns had HL, and 17.1% (37.8% of those with HL) had bilateral HL. The prevalence of bilateral HL was 0.11%, which is comparable with those of previous reports ^(1), (2)^.

As for the results of the diagnostic examinations, the rate of HL among the newborns who were screened in the NICU and that among HN could not be calculated since information on NICU admission or tracking information from the municipalities were not available. In 18 patients who underwent diagnostic examinations, the final results were unknown. The main reasons for an unknown result were a change in the address and loss of contact. Since there was no system to register the results of the diagnostic examination to individual cases across municipalities, the ability of the municipalities to track the cases was limited. Presently, in Kochi Prefecture, a system for hospitals to contact municipalities periodically with the examination results exists, which has made it easier to obtain examination results.

In this study, individual results were not registered in a database for analysis, but the aggregate results from each municipality and the NICU were collected separately, and the referral rates of HN were calculated. The referral rates of HN were only estimated because the municipalities did not distinguish between newborns admitted to the NICU and those not admitted to the NICU. Nevertheless, in the Kochi Prefecture, all premature newborns are admitted to one of the three hospitals with NICUs almost without exception, and the full public expenditure allowed for a high screening rate, making it possible to comprehensively identify the target population. An aggregate analysis of all births in the region would be useful for confirming the screening accuracy and HL prevalence, as we did in this study.

## Article Information

### Conflicts of Interest

None

### Acknowledgement

We would like to express our deepest gratitude to Ms. Nana Ono RN, PHN (Maternity Health and Parenting Support Office, Kochi Prefectural Government) for her efforts in correcting and collecting additional data on the content of the reports from municipalities or hospitals.

### Author Contributions

IF collected all data used in this research and wrote the manuscript. TK and KH gave technical support and conceptual advice. All authors read and approved the final manuscript.

### Approval by Institutional Review Board (IRB)

Ethical approval was waived for this study because personal information was not analyzed. To use internal documents held by the Kochi Prefectural Government, the research protocol was submitted to and approved by the Treatment and Welfare Center, an affiliated facility of Kochi Prefecture (gan-ko-ryoiku-1034, October 9, 2019).
